# In vitro reconstruction of branched tubular structures from lung epithelial cells in high cell concentration gradient environment

**DOI:** 10.1038/srep08054

**Published:** 2015-01-27

**Authors:** Masaya Hagiwara, Fei Peng, Chih-Ming Ho

**Affiliations:** 1 Nanoscience and Nanotechnology Research Center, Research Organization for the 21st Century, Osaka Prefecture University, 1-2, Gakuen-cho, Naka-ku, Sakai, Osaka 599-8570, Japan; 2 Mechanical and Aerospace Engineering Department, University of California Los Angeles, 420 Westwood Plaza, Los Angeles, CA 90095, USA; 3 Innovation instisute, Huazhong University of Science and Technology, Wuhan, China, 430074; 4 Bioengineering Department, University of California Los Angeles, 420 Westwood Plaza, Los Angeles, CA 90095, USA

## Abstract

We have succeeded in developing hollow branching structure in vitro commonly observed in lung airway using primary lung airway epithelial cells. Cell concentration gradient is the key factor that determines production of the branching cellular structures, as optimization of this component removes the need for heterotypic culture. The higher cell concentration leads to the more production of morphogens and increases the growth rate of cells. However, homogeneous high cell concentration does not make a branching structure. Branching requires sufficient space in which cells can grow from a high concentration toward a low concentration. Simulation performed using a reaction-diffusion model revealed that long-range inhibition prevents cells from branching when they are homogeneously spread in culture environments, while short-range activation from neighboring cells leads to positive feedback. Thus, a high cell concentration gradient is required to make branching structures. Spatial distributions of morphogens, such as BMP-4, play important roles in the pattern formation. This simple yet robust system provides an optimal platform for the further study and understanding of branching mechanisms in the lung airway, and will facilitate chemical and genetic studies of lung morphogenesis programs.

Branching morphogenesis is a complex developmental process. Many organs such as the lung airway, kidney, and mammary gland contain branched epithelial tubes^1^,^2^,^3^. The lung airway has a particularly complex branching morphogenesis program, which is required to fill a three-dimensional (3D) space with tubes at high density to optimize their physiological functions^4^,^5^,^6^. The structure of the lung airway consists of thousands of branched arrays and, although the spread from the main shaft appears random, the pattern is identical between individuals of the same species^7^. Many studies have led to the partial elucidation of branching mechanisms; this body of work has identified some of the key morphogens (FGF 10, BMP4, and Btbd7) required for the process^8^,^9^,^10^,^11^,^12^,^13^,^14^,^15^. Nevertheless, a full understanding of the developmental mechanisms that control 3D branching systems is still lacking. This is in large part due to a lack of successful in vitro experiments that can use single type of cells to develop branching structures. Indeed, most studies depend on embryonic tissue culture, which contains not only epithelium cells but also various cell types such as endothelium cells, mesenchymal cells, and fibroblasts. Cell-cell interaction between heterotypic cell culture plays an important role in cell development^16^,^17^,^18^,^19^,^20^,^21^,^22^, but the complex signal communications obscure the contributions of each cell signaling module. Thus, how the morphogens act in what combinations are still poorly understood^23^. Establishing a procedure to develop branching structures from single type of cells in vitro would therefore greatly facilitate research in this area. A previous study reported the development of lung airway branching structures in vitro following co-culture of specific cell lines^24^,^25^ with human umbilical vein endothelial cells (HUVECs). However, there was considerable variation in morphology under these conditions, likely because of differences in transfection efficiency in each cell line used. Thus, there is room for further refinement of in vitro methods for investigating lung branching morphogenesis.

Here we report a simple procedure for the development of 3D branches in vitro using commercially available normal human bronchial epithelial (NHBE) cells. The procedure does not require co-culture with heterotypic cells. We focused on the effect of cell concentration and requirements of spatial distribution in the extracellular matrix (ECM) to evaluate branching morphogenesis. A reaction-diffusion (RD) model was used to explain the branching morphogenesis that occurred at different cell concentration gradients. The result facilitates the identification of key signaling mechanisms required for the development of branching structures.

## Results

### Branching morphogenesis from cell clotting

**Figure 1:**
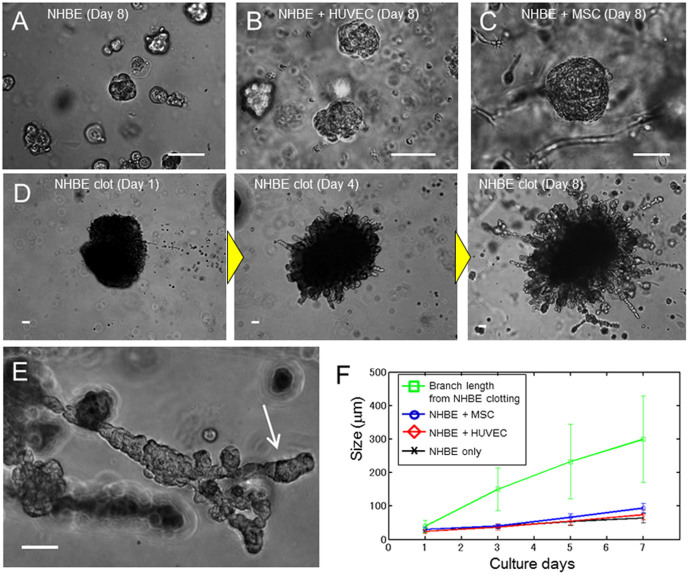
Comparison of lung airway morphogenesis in 3D culture. (A) Phase contrast image of monocultured NHBE cells at day 8. NHBE cells formed spherical colonies, but no branching morphogenesis occurred. (B) Phase contrast image of NHBE cells co-cultured with HUVECs. No branching morphogenesis occurred, but the growth rate of NHBE cells was slightly higher than controls. (C) Phase contrast image of NHBE cells co-cultured with MSCs. No branching morphogenesis occurred. The size of the NHBE cells is double that of the control case at day 8. (D) Phase contrast image of NHBE cell clots in Matrigel at day 1, day 4, and day 8. The clot consisted of 2 × 10^5^ cells at day 1. The branches grew out from the clot at day 4, and the growth rate was much faster than the cultures that were homogeneously distributed. At day 8, the maximum length of branches reached 700 μm and number of branches from the clot was increased. (E) Higher magnification image of branches at day 8. Secondary branching was observed. (F) Quantitation of cell growth rate. Co-culture of NHBE cells increased the growth rate, but the cells grew much faster in NHBE cell clots. The size of branches from cell clots at day 3 were 3 times higher than the cell sizes cultured homogeneously (p = 0.027). Error bars indicate standard deviation (n = 20). Scale bars 100 μm (A–E).

To evaluate the lung airway morphogenesis, we cultured NHBE cells in reconstituted basement membrane (rBM). Matrigel was used as rBM in the following 3D culture experiments. It was reported that co-culture of HUVECs or mesenchymal stem cells (MSCs) is required to make a branching structure of lung epithelium^5^,^24^,^25^,^26^,^27^. Thus, we examined the effects of both heterotypic co-culture of NHBE with HUVECs or MSCs as well as NHBE clotting monoculture on NHBE morphogenesis. When NHBE was homogeneously distributed in Matrigel, cells formed spherical colonies, but no branching was observed (Fig. 1A). On the other hand, when NHBE cells were cultured with HUVECs or MSCs, they formed larger spherical colonies in comparison with homogeneously distributed NHBE monocultures (Fig. 1B and C). Despite this, NHBE cell branching morphogenesis was absent in both cases. In order to observe the effects of cell concentration gradient, we then established NHBE cell clots using fibrin produced from the reaction between fibrinogen and thrombin (Fig. 1D). This procedure causes radial elongation of cells from the clot, and subsequent formation of branching structures. After 5 d, some of the initial branches also began to form secondary branches (Fig. 1E).

**Figure 2:**
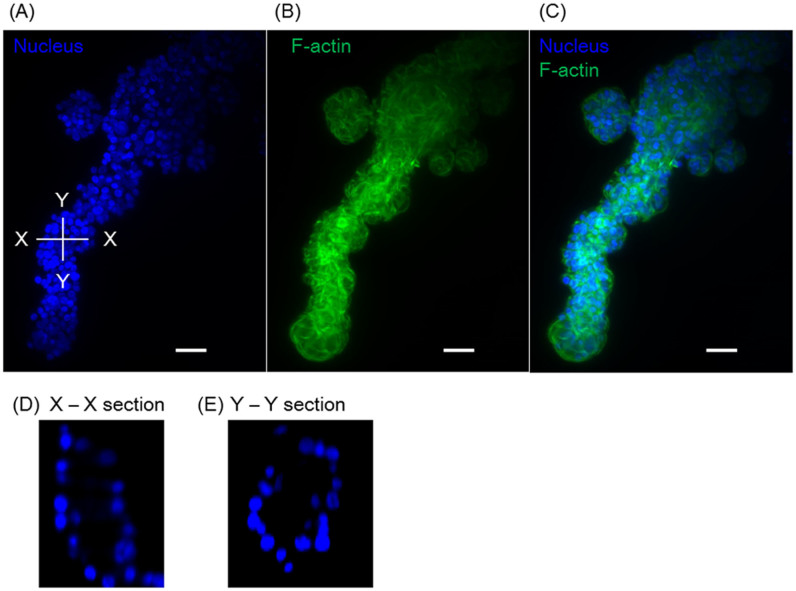
Fluorescent image of branching structures. (A) Nucleus image, (B) F-actin, (C) composite image of (A–B). (D) Cross section of the X-X plane, in which the hollow structure can be observed (E) Cross section of the Y-Y plane. Scale bars 100 μm.

Fig. 1F shows cellular growth over time. Compared to the homogeneously distributed NHBE cell culture, the cells grew larger in the case of co-culture with HUVEC and MSC. This indicates HUVEC and MSC produce morphogens to grow NHBE or stimulated NHBE. In any case, cells derived from the clots grew much faster. From day 3, the branch length was more than 3-fold greater than that observed when NHBE cells were co-cultured with HUVECs and MSCs (p = 0.027), and branch length reached a maximum of 700 μm by day 8. The variation of the branch length from a clot at day 7 was relatively large (100 μm to 700 μm) due to the out-of-round shape of clot but even shorter branch length exceeded size of co-cultured NHBE. Figure 2 shows the fluorescent images of a 3D branching structure derived from NHBE cell clot. Nucleus and F-actin of NHBE were stained to analyze the branching structure. The inside of the branch formed a hollow structure and the branches exhibited tubular patterns. Based on nucleus counting from the 3D image, the developed branch was consist of approximately 70 cells per 100 μm and the tubular was consist of 10–25 cells depending on the sections.

### Effects of cell concentration on branching morphogenesis

**Figure 3:**
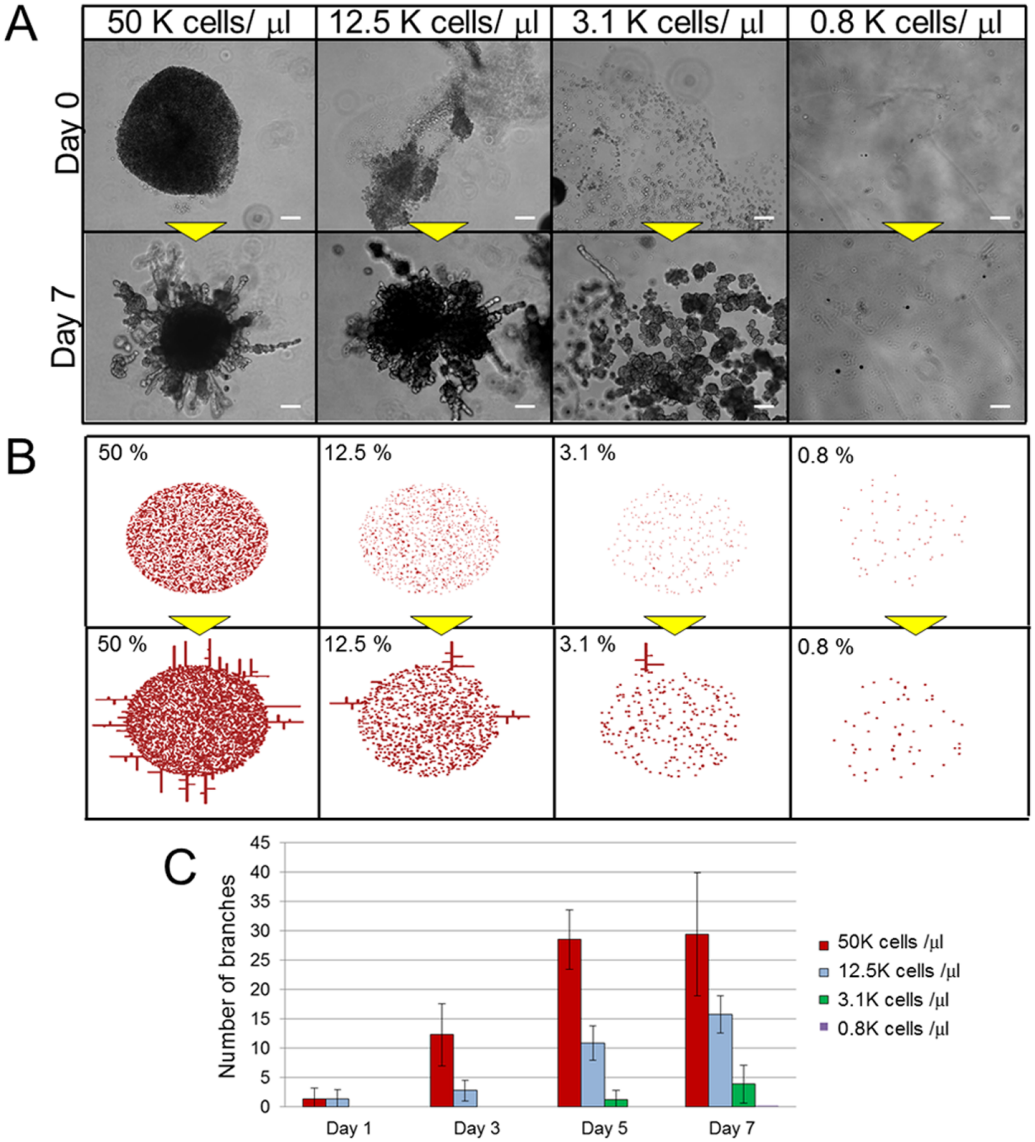
Comparison of lung airway morphogenesis at different cell concentrations. (A) Phase contrast images of NHBE cells with 50 × 10^3^ cells/μl, 12.5 × 10^3^ cells/μl, 3.1 × 10^3^ cells/μl, and 0.8 × 10^3^ cells/μl at day 1 and day 5. The injected cells formed aggregates, and branch formation was proportional to the initial number of cells injected. Scale bar: 200 μm. (B) The initial conditions and the results after 4000 calculation iterations of reaction-diffusion model in various cell concentrations in the center area. The branches were generated in the cases of higher concentration, and branches increased with increasing concentration. Parameters: c = 0.04 ± 5%, μ = 0.12, ν = 0.04, ρ_A_ = 0.014, ρ_H_ = 0.00014, c0 = 0.02, γ = 0.02, ε = 0.087, d = 0.0013, e = 0.1, f = 10, D_A_ = 0.015, D_H_ = 0.18, D_S_ = 0.06 (C) Numbers of branches >100 μm in length. In cases of higher concentrations, the branches were generated from day 1, and the numbers of branches increased rapidly, while branching did not develop (or required more time to develop) at lower cell seeding densities. The number of branches were increased with increasing cell seeding densities in a certain area (R = 0.88, p = 0.0037). Error bars indicate standard deviation (n = 8).

In order to elucidate the mechanism of branching morphogenesis in the context of NHBE cell clots, we first considered the effects of cell concentration, since this parameter is an important determinant of cell growth^28^,^29^. Therefore, one micro-litter of droplet with various NHBE cell concentrations were injected into the center of the Matrigel and observed the morphogenesis (Fig. 3A). At higher concentrations, cells aggregated and formed large clots even in the absence of fibrin and thrombin (Fig. 3A). Subsequently, many branches grew from these clots, and elongated in a manner similar to those shown in Fig. 1D. On the other hand, cells at intermediate density (3.1 × 10^3^/μl) formed many small clusters with their neighbors, and produced fewer branches. At low density (0.8 × 10^3^/μl) cells did not aggregate at all and did not form any branching structures.

Fig. 3B shows the simulation result obtained using an RD model. The RD model enables the computation of large-scale signal communications between cells, and (based on calculations of spatial chemical diffusion and reactions) provides estimates of how specific patterns can be generated by the cells. As an initial condition, calculations were only based on cells distributed in a limited area at the center. Cell concentrations were set as 50%, 12.5%, 3.1%, and 0.8% of the cell distribution area. After 2000 iterations of calculations, branches were generated radially from the cells at the edge of the distribution area, with a higher cell concentration leading to the production of more branches. This is because the relative position of each cell to its neighbor is closer at high concentration, and diffusion of activators stimulates other cells to produce additional activators due to a positive feedback loop. As a result, the total amount of activator increases at higher cell concentrations. The simulation result corresponded well to the experimental results. Fig. 3C shows the number of developed NHBE branches in the experiments with more than 100 μm length over time. At high concentrations (1.25 × 10^4^ cells/μl and 5 × 10^4^ cells/μl), branches developed more rapidly from day 1 when compared to lower concentrations. On the contrary, no branching was observed until day 7 at 3.1 × 10^3^ cells/μl and no branching was observed at 0.8 × 10^3^ cells/μl. Together, these data show that the growth rate of NHBE cells is higher when the initial cell concentration is high, and that this associated with more rapid branching (R = 0.88, p = 0.0037).

### Spatial requirements of branching morphogenesis

**Figure 4:**
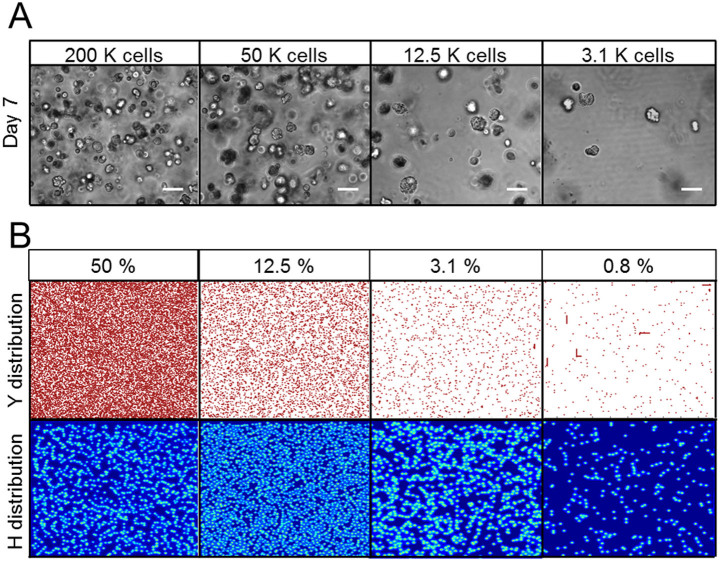
NHBE cell morphogenesis following homogeneous distribution of cells in Matrigels. (A) Phase contrast images of homogeneously distributed NHBE cells cultured with various cell concentrations at day 7. Even when cells were seeded at high concentration, no branching was observed under homogeneous seeding conditions. Scale bar: 100 μm. (B) Result of reaction-diffusion model calculations with various cell concentrations. Cells were distributed homogeneously in a confined space. Y distribution indicates normalized cells positions and size. H distribution indicates normalized inhibitor concentration distributions produced by cells themselves. At higher cell concentrations, the inhibitor was occupied in a confined space and inhibited cell growth. Parameters: c = 0.04 ± 5%, μ = 0.12, ν = 0.04, ρ_A_ = 0.014, ρ_H_ = 0.00014, c_0_ = 0.02, γ = 0.02, ε = 0.087, d = 0.0013, e = 0.1, f = 10, D_A_ = 0.015, D_H_ = 0.18, D_S_ = 0.06.

Next, we examined the spatial requirements for branching morphogenesis. NHBE were filled in culturing area and distributed homogeneously in 100 μl Matrigel with various cell concentrations (Fig. 4A). Cells formed spherical colonies, but no branches were observed at any concentrations at day 7 although branches were developed rapidly with higher cell concentration when cells were confined to a limited area in the Matrigel (as described above). This observation was supported by another RD model after 2000 iterations of calculations (Fig. 4B). Cells were set homogeneously over the calculation area with concentrations of 50%, 12.5%, 3.1%, and 0.8%. Y distribution shows the actual cell positions and H distribution shows the inhibitor concentration distribution over the calculation area. At higher coverage concentration (50%, 12.5%, 3.1%), the inhibitor filled the entire space, since its diffusion rate is much faster than that of the activator^30^,^31^. Thus, cells were prevented from developing branching structures. On the other hand, while cells seeded at lower coverage (0.8%) had more space to grow, the amount of activator they encountered locally was insufficient to produce branching; this is because the distance between neighboring cells was too far. This indicates the importance of proximity for activator-induced branching, as some of the cells were accidentally close to each other, and thus elongated in small spaces.

### Morphogen expression on developed branches

**Figure 5:**
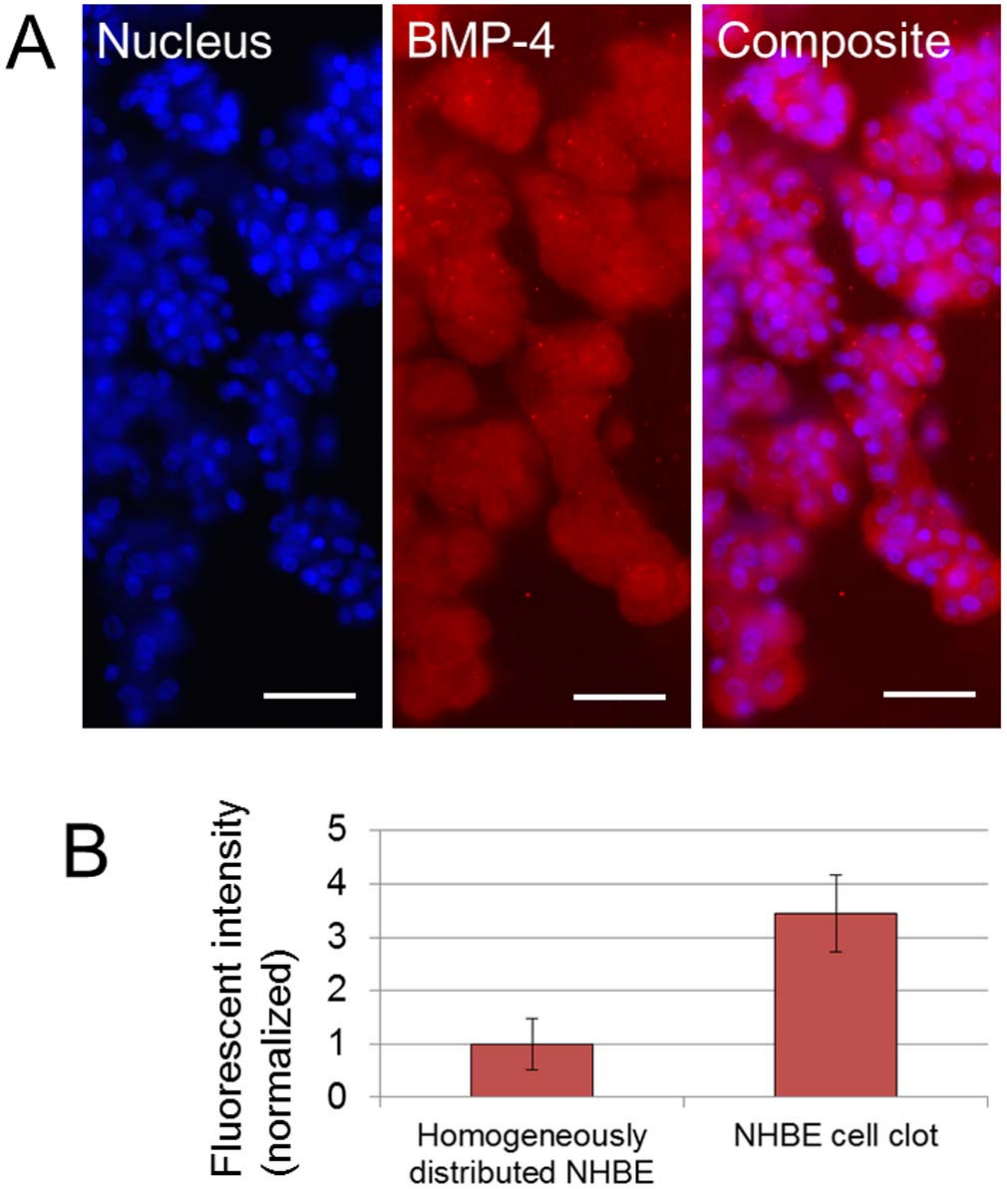
Fluorescent imaging result of BMP-4 expression on the branch. (A) Fluorescent images of nucleus and BMP-4 on developed branches from NHBE clot. BMP-4 expression was observed over the cell clot and branches. Scale bars 100 μm. (B) Comparison of fluorescent intensity of BMP-4 expression from homogeneously distributed NHBE and from NHBE cell clot with branches after day 7. The intensity from cell clot is higher than from homogeneously distributed NHBE (p = 0.014). Exposure time 300 ms. (n = 6).

It is considered in many studies that one of the potential activator, inhibitor and substrate for the lung airway are bone morphogenetic protein 4 (BMP-4), matrix GLA protein, and fibroblast growth factor 10 (FGF10) respectively^15^,^32^,^33^,^34^,^35^. Fig. 5A shows the fluorescent image result by applying anti-BMP-4 antibody to the developed branches from NHBE clot. BMP-4 was expressed over the branches as well as the clot area. Next, fluorescent intensity of BMP-4 expression was measured in both cases of NHBE clots and homogeneously distributed NHBE culture after day 7 (Fig. 5B). The exposure time of UV light was set as 300 ms and the maximum fluorescent intensity was measured. Compared to the homogeneously distributed NHBE cell culture, the fluorescent intensities from cell clots were higher (p = 0.014). These experimental results indicate that cell clot produce much more activator (BMP-4) in a certain area.

In the RD simulation, the activator concentration becomes higher when cells are close to each other owing to the positive feedback of the activator. And when the activator concentration exceeds a certain value, the branches will be developed. Above experimental results are corresponds with the simulation results.

## Discussion

**Figure 6:**
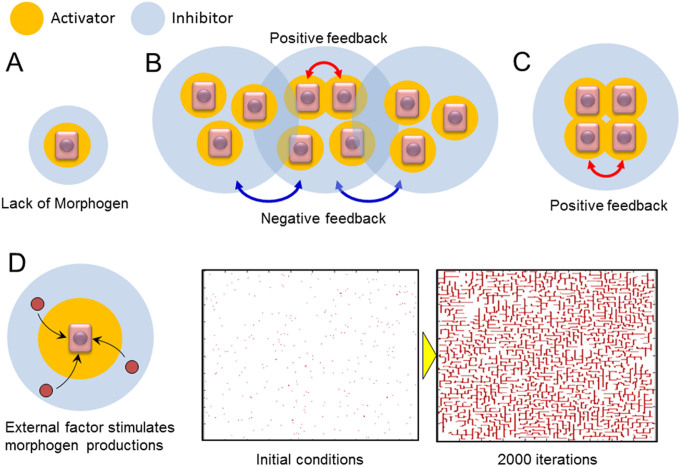
Model of branching development requirements in lung airways. (A) Low cell concentration scenario. The local morphogen concentration is insufficient to promote branching structure development. (B) High cell concentration, but low gradient scenario. Neighboring cells increase localized morphogen production, but long-range inhibitors from other cells prevent branching development. (C) High cell concentration gradient scenario. Cells in neighbors increase morphogen productions, and the inhibitors produced by other cells do not interrupt branching morphogenesis. (D) Model and the reaction-diffusion simulation result under conditions in which cell concentration is low and an external factor is applied to stimulate morphogen productions. The initial cell concentration in the simulation was 0.8% over the calculated space and 2000 calculation iterations were performed. The results indicate that the branching structure will be produced even in homogeneously distributed cell cultures when cellular production rate of morphogen is increased by external stimulation such as co-culturing with heterotypic cells. Parameters: c = 0.04 ± 5%, μ = 0.12, ν = 0.04, ρ_A_ = 0.03, ρ_H_ = 0.0003, c_0_ = 0.02, γ = 0.02, ε = 0.2, d = 0.0013, e = 0.1, f = 10, D_A_ = 0.015, D_H_ = 0.18, D_S_ = 0.06.

The experimental and simulation data described herein reveal that both cell concentration and space for growth are required for productive NHBE cell branching in vitro. In general, the diffusion rate of inhibitors is much faster than that of activators in cases where cells self-organize into patterns. Thus, activators are able to stimulate the production of morphogens in proximal cells, while inhibitors block morphogen production preferentially in more distal cells^36^. When the cell concentration is low, morphogen concentration is also low; this occurs since the activator production near the cells are not enhanced, which thus precludes the formation of branching structures (Fig. 6A). However, high cell concentration alone is also insufficient to initiate branching structure development. When cells are diffusely and randomly spread, long-range negative feedback dominates the culture. Activators produced by neighboring cells enhance activator production and initiate a positive feedback loop; however, long-range inhibitors from surrounding cells counteract this cell activation and prevent them from executing a branching morphogenesis program (Fig. 6B). On the other hand, when cell concentration is high in a restricted area and the surrounding area is completely free of cells (i.e., the cell concentration gradient is high), the effect of positive feedback is amplified since less inhibitory signals are present (Fig. 6C). At the boundary of cell cluster and the free space, high morphogen gradient exists. It is known that the interface across the sharp morphogen gradient is not stable^41^, which is the fundamental mechanism of generating the branch structures. The interface instability will make the smooth density forefront develop corrugations, which the corrugated front will induce the cells expanding non-uniformly into the free space and eventually branches are produced. Under these conditions, airway cells begin to produce branching structures.

Branching of some lung airway cell lines has been achieved by co-culturing them with HUVECs^24^,^25^. In this scenario, it is likely that the HUVECs stimulate airway cells to increase the production rate of morphogens (Fig. 6D). Here, we validated this model by re-testing an RD simulation with the higher production rate of morphogens. The parameters related to morphogen production and consumption by cells, such as production rate of activator and inhibitor secreted by cell, substrate consumption rate by cells, were increased in the simulation while other conditions were same as the case of 0.8% concentration in Fig. 4B. Then many of branching structures were observed in the calculation area while no branching occurred when morphogen production and consumption rate was low (Fig. 4B). Thus, airway cells can develop branching structures if the production rate of morphogen exceeds a critical threshold. Until the present study, however, there have been no experimental systems capable of recapitulating branching lung epithelial structures using just one primary cell type. Our simple system will allow research groups to identify critical combinations of morphogens and their inhibitors that govern the process of branching morphogenesis.

### Experimental Methods and Simulations

#### Three-dimensional culture

NHBE cells and HUVECs were both from Lonza (Walkersville, MD). MSCs were isolated as described previously^37^. The growth factor-reduced reconstituted basement membrane, Matrigel (Corning Incorporated; Corning, NY) was used for 3D culture experiments. Cells were seeded into 300 μl Matrigel in suspension and gently mixed with a pipet. They were then added into 24-well plates and incubated for 25 min at 37°C to facilitate gelatinization. One milliliter of bronchial epithelial growth medium (BEGM; Lonza; Walkersville, MD) was added to each well and the medium was changed every other day. For co-culture experiments, 5 × 10^3^ NHBE cells and 1.0 × 10^6^ HUVECs or MSCs were seeded into Matrigel. For experiments with various concentrations of cells seeded in a limited area (Fig. 3), cell concentration was adjusted after centrifugation, and 1 μl of this suspension was injected with a pipet into the center of Matrigel before gelatinization.

#### Clotting experiments

In order to investigate the effect of high cell concentration gradient, NHBE cell clotting was made in a fibrin gel clot. Human fibrinogen (Sigma-Aldrich; St. Louis, MO) and bovine plasma thrombin (Sigma-Aldrich; St. Louis, MO) were prepared as 2 mg/ml and 2 U/ml solutions, respectively, in 1% bovine serum albumin (BSA). Then, 2.5 μl fibrinogen solution and 2.5 μl thrombin solution were gently mixed on a glass slide with 2 × 10^5^ NHBE cells and the mixture was incubated for 10 min at 37°C. After NHBE cell clots were made, 150 μl of Matrigel was placed on each well of a 24-well plate as a base gel; the NHBE cell clot was transferred to the top of the Matrigel, after which a further 150 μl of Matrigel was added on top of the NHBE cell clot. After 25 min incubation at 37°C, the NHBE clot was cultured in 1 ml of BEGM.

#### Fluorescent staining

Nucleus and actin filament of NHBE were stained to visualize the branching structures. For fixation, 4% paraformaldehyde (Electron Microscopy Science; Hatfield, PA) was applied to Matrigel at room temperature for 20 min. Cells were permeabilized with PBS containing 0.5% Triton X-100 for 10 min at 4°C followed by three washes of 10 min each with PBS containing 100 mM glycine. Then, cells were incubated with 10% goat serum in IF-buffer (0.2% Triton X-100; 0.1% BSA and 0.05% Tween-20 in PBS) for 60 min at room temperature for a primary block. As a secondary block, cells were incubated with 1% goat anti-mouse immunoglobulin G and 10% goat serum in IF-buffer for 40 min at room temperature. Cells were then incubated overnight at 4°C with anti-BMP4 antibody (Sigma-Aldrich; St. Louis, MO) diluted by the blocking solution followed by three washes of 20 min each with IF buffer. Alexa fluor 555 (Lifetechnologies; Grand Island, NY) was applied as secondary antibody and incubated for 2 hour followed by three washes of 10 min each with PBS. For nuclear staining, cells were incubated with 300 nM DAPI in PBS for 20 min at room temperature, followed by three rinses of 20 min each by PBS. For staining actin filaments, cells were incubated for 20 min at room temperature with Alexa fluor 488 phalloidin (Lifetechnologies; Grand Island, NY), followed by three rinses of 20 min each by PBS.

#### 3D imaging

Lightsheet microscopy Z.1 (Carl Zeiss Microscopy GmbH, Jena Germany) was used to obtain large-scale 3D images of branching structures. The sample contained in Matrigel was covered by 1% of agar gel with PBS and transferred to fluoropolymer tube (inner diameter: 2.4 mm, outer diameter: 3.2 mm). 20 magnification lens of W Plan Apochromat (numerical aperture: 1.0) was used as detection lens. The step size of z direction was 2.5 μm and 83 slices images were taken. The images were reconstructed to 3D shape by ZEN software (Carl Zeiss Microscopy GmbH, Jena Germany).

#### Reaction-Diffusion Simulation

The Reaction-Diffusion (RD) model, which was initially proposed by A. Turning in the 1950s, mathematically express how a morphogen spatially diffuses or reacts to other morphogens in order to generate specific patterns by computing large-scale signal communications^31^. Subsequently, H. Meinhardt extended the RD model was extended to incorporate elements such as the global network and branching^38^,^39^,^40^. In order to elucidate the effect of cell concentration gradients on branching development, we carried out a simulation based on the model described by Meinhardt^40^. The differential equations are as follows, 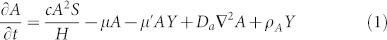

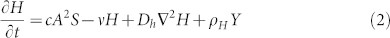

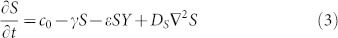

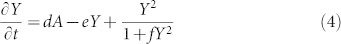
 The four variables used in this model are an activator concentration (A), an inhibitor concentration (H), a substrate chemical (S), and a biological marker (Y). Activator A increases the morphogens (A and H) production, while inhibitor H blocks morphogen production. Substrate S is required for the production of morphogens and is consumed by cells. A biological marker Y is a fixed value when it exceeds a specific threshold. At this point, Y is not affected by morphogen concentration, and the cells are fixed at that position. The other characters are experimental constants.

In order to express the starting cell positions, the initial Y values were set above the critical threshold, at a point where Y is stable (Y = 1), at the randomly selected position in a certain area; in other areas Y was set as 0. The parameters for each simulation are as follows: for Figs. 3 and 4, c = 0.04 ± 5%, μ = 0.12, ν = 0.04, ρ_A_ = 0.014, ρ_H_ = 0.00014, c_0_ = 0.02, γ = 0.02, ε = 0.087, d = 0.0013, e = 0.1, f = 10, D_A_ = 0.015, D_H_ = 0.18, D_S_ = 0.06; for Fig. 5, c = 0.04 ± 5%, μ = 0.12, ν = 0.04, ρ_A_ = 0.03, ρ_H_ = 0.0003, c_0_ = 0.02, γ = 0.02, ε = 0.2, d = 0.0013, e = 0.1, f = 10, D_A_ = 0.015, D_H_ = 0.18, D_S_ = 0.06. By calculating the partial differential equations, one can simulate the mode of cell growth by following the spatial distribution of Y values.

## Author Contributions

M.H. and C.M.H. conceived and designed the three-dimensional culture experiments and simulations; M.H. performed three-dimensional culture experiments and simulations; F.P. performed three-dimensional visualization of branched structure; M.H. and C.M.H. wrote the paper.
